# Migraine and Diet

**DOI:** 10.3390/nu12061658

**Published:** 2020-06-03

**Authors:** Parisa Gazerani

**Affiliations:** Laboratory of Molecular Pharmacology, Department of Health Science and Technology, Faculty of Medicine, Aalborg University, 9220 Aalborg East, Denmark; gazerani@hst.aau.dk

**Keywords:** diet, migraine, triggers, nutrition, disease modification, epigenetics, headache

## Abstract

Migraine is characterized by recurrent attacks of disabling headaches, often accompanied by sensory and motor disturbances. Clinical manifestations of migraine are influenced by dietary behaviors and dietary elements. Several dietary triggers for migraine have been identified, leading to the definition of strategies such as elimination diets, ketogenic diets, and comprehensive diets, mainly to help prevent migraine. Although inconsistency is present in the literature and no consensus exists, the available data are promising in supporting beneficial dietary interventions for some migraine patients. Several factors influence the net outcome, including age, sex, genetics, and environmental factors. Advancement in understanding the underlying mechanisms of migraine pathogenesis and how dietary factors can interfere with those mechanisms has encouraged investigators to consider diet as a disease-modifying agent, which may also interfere with the gut–brain axis or the epigenetics of migraine. Future work holds potential for phenotyping migraine patients and offering personalized recommendations in line with biopsychosocial models for the management of migraine. Diet, as an important element of lifestyle, is a modifiable aspect that needs further attention. Well-designed, systematic, and mechanism-driven dietary research is needed to provide evidence-based dietary recommendations specific to migraine. This narrative review aims to present the current status and future perspective on diet and migraine, in order to stimulate further research and awareness.

## 1. Introduction

Migraine is a common neurological disorder that is accompanied by disabling headaches and a plethora of somatosensory and transient motor disturbances [1]. Migraine occurs in episodic or chronic forms, with and without aura [2]. The possible mechanisms underlying migraine have been investigated [3] and, generally, it is accepted that the activation of the trigeminovascular system (e.g., by circulating pro-inflammatory substances and oxidative state) is involved [4]. Migraine is a multidimensional and complex disorder [5], which is influenced by genetic and environmental factors [4,6]. Attempts have been made to find preventive and treatment strategies that are efficient and safe [7]. However, the available medications [8] or non-drug strategies [9] remain only partially beneficial. Therefore, the control of migraine remains challenging. Researchers have attempted to develop lifestyle modification strategies to prevent and treat headaches [10]. In this line, the role of dietary triggers has been recognized [11], leading to strategies for diet therapy for headaches, including migraine [12,13]. Precision nutrition is an emerging field in the design of personalized nutritional solutions for several disorders, such as metabolic syndrome [14,15]. Interestingly, migraine has been proposed as a disorder linked to metabolism [16] or a metabolic endocrine disorder [17]. The contribution of dietary compounds to headache pathogenesis has been recognized and, based on this, an elimination diet strategy has been introduced in the field [12,18,19]. Generally, it is accepted that migraines are sensitive to diet and that some dietary ingredients trigger migraine attacks. Long lists of potential dietary triggers exist, but controversy has remained in the field. Chocolate, citrus fruits, nuts, ice cream, tomatoes, onions, dairy products, alcoholic beverages, coffee, caffeine, monosodium glutamate (MSG), histamine, tyramine, phenylethylamine, nitrites, aspartame, sucralose, and gluten have been noted in the literature [18,19,20]. The response of a headache patient to a given dietary trigger may depend on the amount and timing of exposure, among other factors [18,19]. High amounts of some food or ingredients may be necessary to trigger a headache, as evidenced by studies involving aspartame or MSG. Some food may trigger headache, while others may cause headache upon withdrawal (e.g., caffeine). Some food or ingredients can trigger headache only in subgroups of patients, such as those with celiac sprue, or in those with specific immunological responses to food, such as those positive for IgG antibodies [18,19]. Therefore, the identification of dietary triggers can be challenging. Food diaries and specific serological testing have been used to identify triggers in individual patients. As genetic factors also play a role and some individuals are more susceptible to the effects of different food, food ingredients, or beverages, the profiling of patients might be helpful to subdivide the heterogeneous migraine population. If dietary triggers can be identified precisely and in an unbiased manner, avoiding those triggers would consequently prevent migraine in affected individuals. This is the basis of elimination diet strategies, but there seem to be controversial findings in the literature. The lack of comprehensive and controlled studies may have caused this controversial outcome. In addition, some comprehensive diets have been introduced for migraine, and the role of probiotics in the modification of gut microbiota in migraine patients has emerged. On the other hand, migraine is a multiphase disorder [21], including a disabling and recurrent headache phase. Some studies using dietary strategies for migraine have considered dietary effects on other symptoms of migraine (e.g., sensitivity to light, noise, and smells, nausea and vomiting, upset stomach, loss of appetite, fatigue, and dizziness) and not on headache *per se*, which is an open area for further investigation. Most likely, a multimodal approach [22], including dietary interventions, may increase the quality of life in a large number of patients with migraine.

## 2. Elimination Diets

Elimination diets require the identification of provocative dietary ingredients and their subsequent elimination. To identify triggering dietary elements [18], a personal approach might be taken, where an individual notices a high frequency of headache or migraine upon exposure to a dietary trigger and, consequently, avoids it. This approach might be biased, due to the variable abilities of observers to recognize true associations. Dietary triggers do not necessarily point to a food allergy. A food allergy can be defined by the activation of antibodies in response to specific food, ranging from mild to serious reactions. To identify food allergies, serological testing can be employed. Food intolerances are more common than food allergies. Therefore, it is important to distinguish between a food allergy and a food trigger for migraine. A complete food diary noting the presence or absence of migraine for assessment by physicians can be useful, from a practical point of view, but food diaries are not problem-free [18]. Exposure to a given food may not always trigger a headache, and the amount of food or the time of exposure might largely influence the outcome [18]. In some cases, there is a gap in time between the consumption of a food trigger and the development of a migraine. The important factor here is to recognize that a complex non-linear system [23] might be involved, and looking for a simple cause–effect relationship might be misleading, when considering food triggers of migraine. A number of other triggers, apart from food (e.g., stress, weather conditions, and dehydration), may initiate the migraine process and must be considered as extra inputs into this non-linear system. As defining an association between food and migraine is challenging, setting up a threshold has been proposed [18]. According to the literature, a food might be considered a trigger if headache occurred in ≥50% of instances within one day of exposure [24]. Multiple trigger factors may be present in one patient, and the identification of a single trigger might be difficult, especially as some factors may potentiate each other. In addition, some food is complex and contains many ingredients; hence, it is hard to identify one specific ingredient as the trigger. Using electronic diaries as part of an app in which it can be determined statistically whether headache is associated with some dietary components might be helpful [18]. The downside of an elimination diet is the long-term negative effect of undernutrition—a form of malnutrition—which is characterized as the inadequate intake of protein, energy, and micronutrients and may result in disorders including psychological problems or infection [25,26].

## 3. Migraine Diets

The idea of specific diets for migraine has been developed alongside elimination diets. Several types of diets have been proposed to be beneficial for migraine. Many of these diets are not mechanism- or evidence-driven, but have been proposed to act through a variety of mechanisms; for example, acting on serotoninergic dysfunction, neuronal excitability, the presence and concentration of substances with a role in migraine pathogenesis (such as calcitonin gene-related peptide (CGRP), nitric oxide (NO), adiponectin, and leptin), brain mitochondrial function, neuroinflammation, hypothalamic function, and platelet aggregation [12]. The rationale must also consider underlying comorbid disorders or even subtypes of migraine (e.g., migraine with aura). To reach the optimal response, considering diets with proven efficacy or a combination of dietary interventions might be useful.

A recent literature review [12] found that ketogenic, high-folate, low-fat, modified Atkins, and high omega-3/low omega-6 diets demonstrated beneficial effects. The Mediterranean diet has also been mentioned, but with fewer available data [12]. Ketogenic and modified Atkins diets have been proposed to promote neuroprotection, improve mitochondrial function, compensate for serotoninergic dysfunction, decrease CGRP levels, and suppress neuroinflammation [12]. Ketogenic diets lead to the elevation of ketone bodies, which have recently been found to be beneficial in migraine prevention [27]. Ketone bodies act on mitochondrial functioning, oxidative stress, cerebral excitability, inflammation, and the gut microbiome [27]. A low glycemic diet might also be useful in migraine. This diet has been proposed to lower the inflammatory state [12]. A balance between the intake of omega-6 and omega-3 fatty acids has also been suggested to reduce inflammatory responses, enhance platelet function, and regulate vascular tone. Therefore, a dietary strategy reducing omega-6 and increasing omega-3 fatty acid intake may prove beneficial for migraine [12].

Sodium levels have been shown to be higher in the cerebrospinal fluid of patients with migraine than in controls, particularly during a headache attack [28]. However, the effects of a low-sodium diet have been shown to be dependent on several factors, which should be evaluated before considering it a beneficial diet for migraine. While a low-sodium diet has been shown to be protective for elderly individuals [29], in a young female population without hypertension and with a low-to-normal body mass index (BMI), a high-sodium diet was shown to be beneficial [30]. Therefore, sodium intake should be tailored to specific patient populations. For example, a diet low in sodium may be appropriate for patients with vascular risk factors such as hypertension, whereas a high-sodium diet may be appropriate in patients with comorbidities such as postural tachycardia syndrome or in those with low blood pressure or low BMI [10].

## 4. Epigenetic Diet

A diet-modifying strategy has been discussed that by adding certain dietary compounds with specific mechanisms of action, one can potentially interfere with disease pathogenesis [31] (e.g., cancer [32]). These types of diets target specific cellular structures (e.g., mitochondria) and molecules (e.g., DNA). Based on this strategy, in 2011, Hardy and Tollefsbol introduced the term “epigenetic diet” [33] to explain that environmental factors such as dietary components can interfere with the epigenetic profile of affected patients and, hence, may be beneficial for the prevention of diseases. This was first considered for cancer prevention [32], but the concept has emerged in other health-associated fields. Rationally, one can consider that diet can alter the epigenetic profile of consumers—for example, in cancer—if aberrant alterations of specific transcription factors occur [34]; therefore, specific diets normalizing those alterations may prevent cancer. However, the underlying mechanisms of such modifications at the cellular and molecular level of the epigenetic profile remain less investigated. In theory, for migraine, such an intervention would mean that a dietary component can block mechanisms underlying migraine or promote prevention mechanisms. This assumption has raised a question on the existence and potential benefits of an epigenetic diet for migraine [26]. This hypothesis has mainly been formed following recent advanced studies in the pathogenesis of migraine with a focus on epigenetics [35,36], where aberrant DNA methylation has been found to be associated with the occurrence of migraine [37]. In several genes, it has been reported that their methylation might be associated with migraine, including SH2D5 (SH2 domain containing 5) [38]; *COMT* (catechol-O-methyltransferase) [39]; *ZNF234* (zinc finger protein 234) [39]; *SOCS1* (suppressor of cytokine signaling 1) [39]; *SLC2A9*, *SLC38A4*, and *SLC6A5* (solute carrier family 2, 38A, and 6A members 9, 4, and 5, respectively) [40]; *DGKG* (diacylglycerol kinase gamma) [40]; *KIF26A* (kinesin family member 26A) [40]; *DOCK6* (dedicator of cytokinesis 6) [40]; *CFD* (complement factor D) [40]; *RAMP1* (receptor activity modifying protein 1) [41]; and *CGRP* [42].

Folate, which is involved in DNA methylation and has previously been shown to be beneficial in migraine, has captured further attention in the context of an epigenetic diet for migraine [26,43]. Recently, it has been proposed that defining a diet, which can target DNA methylation—for example, a folate-rich diet—could potentially provide a future direction for migraine-related epigenetic dietary studies [26,44]. Further investigation is required to provide evidence on the potential dietary components, which may interfere with the epigenetics of migraine [37,45,46]. Most likely, this approach will require the epigenetic profiling of migraine patients [46] before an epigenetic diet for migraine can be defined. In addition, one must consider that other elements of the cellular epigenetic profile, such as histone modification and the action of non-coding RNAs (ncRNAs) [36], in addition to DNA methylation, can be modified by dietary components, resulting in the alteration of proteins or RNA products. This field is open for investigation, but issues related to bioavailability and final biological effects, synergistic or inhibitory effects when used alone or in combination with other substances, tissue-specific absorption, individual features of consumers, and the role of sex and other body characteristics must be taken into consideration, as these factors alone or in combination can alter the outcome [26,37]. In addition, migraine comorbidities, such as depression and epilepsy, must be taken into account. Another important point is that, at present, several chemicals targeting the epigenome have been accepted as epigenetic drugs [47]. For example, valproate has long been used for the treatment of epilepsy and has been shown to be effective in a number of migraine patients. Valproate is a histone deacetylase (HDAC) inhibitor [48], and evidence shows that it also triggers DNA demethylation [49]. Hence, it has been considered an epigenetic drug [46]. However, epigenetic drugs are different from epigenetic diets. For example, ketosis was reported to alter cellular functions through epigenetic mechanisms. On the other hand, the ketogenic diet has been considered a rapid and effective diet for migraine. Drawing a link between these two observations is not easy, as it is still not clear which epigenetic mechanisms are altered by ketosis and which components of the ketogenic diet act on the epigenome [26]. Therefore, the advantages of an epigenetic diet over epigenetic drugs must be determined first.

Folate supplements are available in the form of folic acid, folinic acid, or 5-methyltetrahydrofolate (5-MTHF) [50]. Studies have shown that folic acid supplementation is beneficial for migraine [51]. Folate is required for providing a methyl group for DNA methyltransferase to methylate DNA (i.e., DNA methylation). In relation to migraine, it has been suggested that a polymorphism in MTHFR (methylenetetrahydrofolate reductase) could result in a homocysteine increase in plasma. The available literature [52] has suggested that elevated plasma levels of homocysteine are associated with an increased risk of migraine. The production of homocysteine requires folate and vitamins B6 and B12. A deficiency in these elements results in DNA hypomethylation, which has been hypothesized to trigger migraine [53]. Collectively, folate is essential for DNA methylation, and its presence in the diet has been reported to exert a beneficial effect on migraine. However, it remains to be determined whether dietary folate can be attributed to changes in DNA methylation or to other alterations in the epigenetic profile. Therefore, investigators have suggested that caution must be taken into account for the application of “epigenetic diet” terminology and for the classification of some diets as “epigenetic diets” (e.g., diets fortified with folate), which might be beneficial for migraine [26].

## 5. Gut–Brain Axis and Probiotics

Several studies have shown different gastrointestinal diseases to be associated with migraine (reviewed in [54]). Migraine is often accompanied by gastrointestinal symptoms, including nausea, vomiting, dyspepsia, and bowel disturbances [55]. A link has also been found that headaches occur at a higher rate in patients with gastrointestinal disorders [56]. Abdominal migraine is an entity affecting children [55]. Studies have also suggested that migraine is associated with inflammatory bowel disease and celiac disease [57].

Recently, the concept of a gut–brain axis, which explains a bidirectional relationship between the gastrointestinal system and the central nervous system [58], has emerged in several medical fields. It has been proposed that cross-talk between the gut and brain may impact several neurological [58] and behavioral [59] disorders. The modulation of the gut microbiota has, consequently, been proposed to treat or prevent those disorders. Evidence in the literature has demonstrated that the application of probiotics might be useful for some neurological disorders, such as Parkinson’s disease [60]. Evidence, however, is limited for migraine, and large-scale randomized, placebo-controlled studies are required to evaluate whether the gut microbiota is involved in migraine pathogenesis [61] and whether probiotics are efficient and safe for patients with migraine headaches [60]. In addition, if the gut–brain axis can influence migraine, the underlying mechanisms remain unexplored. It has been proposed that, due to increased intestinal permeability, pro-inflammatory substances may reach the trigeminovascular system and trigger migraine-like attacks [62]. This theory is in line with previous findings demonstrating that a link exists between migraine headache and various inflammatory diseases, including allergies [63] and asthma [64]. Generally, multiple factors, such as inflammatory mediators (IL-1β, IL-6, IL-8, and TNF-α), the gut microbiota profile, monoamines (e.g., serotonin, dopamine, and norepinephrine), stress hormones, and nutritional elements [61], have been considered to play a role. Alteration in the gut microbiota by probiotics has demonstrated beneficial effects, and certain probiotics—mainly strains of *lactobacilli* and *bifidobacteria*—could prove useful to increase the integrity of the gut epithelial barrier [65]. The mechanisms underlying the potential effects of probiotics in neurological disorders are not well understood, but they have been proposed to be multidimensional, resulting in the maintenance of gut barrier integrity, permeability, and function. Studies investigating probiotics for migraine are limited and often mix the application of probiotics with other components. A review of 70 trials of probiotics in migraine reported a lack of consistency in the results, prohibiting the use of meta-analysis to draw a conclusion on the beneficial effects of probiotics for migraine [66]. Generally, no severe side effects have been reported, but mild symptoms (including constipation, nausea, bloating, and diarrhea) are present with some probiotics [67]. Overall, evidence suggests that alterations in the gut microbiota based on dietary changes and possibly inflammation reduction can have a significant impact on migraine. It has also been proposed that migraine may be improved by dietary approaches with beneficial effects on the gut microbiota and gut–brain axis, including appropriate consumption of fiber, low glycemic-index diet, supplementation with vitamin D [68] and omega-3 fatty acids, low-fat vegan diet, gluten-free diet, probiotics, and weight-loss diets [61]. The latter is based on studies that have demonstrated an association between obesity and migraine and clinical improvements after weight loss [61,69,70,71,72]. For example, Verrotti et al. [72] evaluated obese adolescents before and after a program involving diet, physical activity, and behavioral therapy. They observed a decrease in weight, BMI, waist circumference, the frequency and intensity of migraine attacks, the use of acute medication, and the Migraine Disability Assessment (MIDAS) score. However, some studies [73,74] in adults yielded no to minimal effects. Therefore, obesity control and weight loss programs may exert different outcomes in different subpopulations of migraine patients, depending on several factors, including age and sex. A recent paper [75] presented an overview of the strength of existing evidence for food-based dietary interventions for migraine and called for high-quality dietary and microbiome research in migraine, both to substantiate the hypothesized relationships and to build up evidence-based information regarding the impact of nutritional interventions on migraine.

## 6. Conclusions

Several factors, including internal (e.g., genetic and epigenetic) and external (e.g., culture, ethnicity, geography, and eating behaviors) factors, play important roles in determining migraine triggers [76]. Even though it is known that dietary triggers influence migraine, the underlying mechanisms remain less explored [27]. Self-control elimination diets are popular; however, the total avoidance of certain substances in these diets [10] (e.g., gluten-free, tyramine-free, or antihistamine diets) might cause malnutrition. Therefore, the selection of an appropriate diet and obtaining correct dietary counseling from physicians and dietitians are recommended. This is to ensure the biopsychosocial well-being of migraine patients, as it has been reported that strict food avoidance may result in stress and poor quality of life [77]. Currently, the ketogenic diet (high fat, low carbohydrate) [78,79] has shown promising results, potentially influencing several pathophysiological mechanisms (e.g., glucose transport, mitochondrial function, oxidative stress, cerebral excitability, cortical spreading depression, inflammation, and the gut microbiome). Supplementation with exogenous ketone bodies as a potential preventative strategy for migraine has also been proposed [80]. Increased fatty acids, amino acids, supplementation with medium-chain triglycerides [81], or other dietary changes, as well as the alteration of the microbiome, have been considered elements for modifying the disease. To achieve the best outcome, the identification of both the phenotypic characteristics of patients and the current reliable biomarkers of diseases has been proposed [82]. With advancement in omics technologies and important steps taken toward personalized medicine [35], an individualized approach in dietary recommendations for migraine patients sounds reasonable [27]. In this line, a balance between trigger avoidance and coping strategies, based on the needs of an individual patient, must be taken into account. Considering the age and sex of patients and whether other types of headaches (e.g., chronic daily headache, cluster headache, or tension-type headache) are influenced by diet, seems crucial.

Research on the microbiome and migraine is still young, and further investigation is required before recommending a dietary intervention intended to change the microbiome for preventing migraine [75]. However, a similar approach might be useful here for the determination of individual differences toward personalized nutritional recommendations. One should also consider the burden of following any diet recommendations. In addition, complexity in this area cannot be neglected for both diet-related comorbidities (e.g., obesity, diabetes, and cardiovascular disease) and migraine-related comorbidities (e.g., depressive disorders). Furthermore, diet is a component of lifestyle and is most likely associated with other lifestyle factors, such as sleep, smoking, and exercise. Vitamins, supplements (e.g., magnesium, riboflavin, coenzyme Q10), and herbal preparations (e.g., feverfew and butterbur) as nutraceuticals have long been considered for migraine [83,84]. These agents might be beneficial, but they may also exert side effects, and patients must be aware of their potential toxicities and interactions with other dietary elements or drugs [84]. For example, cases of hepatotoxicity in relation to butterbur have emphasized a need for caution and the monitoring of safety in relation to nutraceuticals [13,85]. Primary care physicians have recently provided best-practice recommendations called SEEDS (Sleep, Exercise, Eat, Diary, and Stress) to help migraine patients by modifying their lifestyle components [10]. Examples of clinical approaches are present in the literature [86] to address dietary recommendations and plans for migraine patients.

## 7. Future Perspectives

Moving forward, research is needed to identify patterns of dysbiosis occurring in persons with migraine, in order to establish a therapeutic target for future trials [75]. Available probiotic trials have provided mixed results. It would be wise to follow the lessons learned from probiotic research in other areas, where the selection and specificity of probiotic strains are essential, including probiotic viability in supplements used in the research and detailed information including background diet [87]. We still do not know whether being a responder or not in response to dietary interventions is due to identifiable microbial profiles. In addition, one needs to consider that the relationship between diet and migraine is not unidirectional, and research needs not only to investigate the influence of diet on migraine, but also the physiological mechanisms of migraine that can potentially influence the choice of diet [88,89,90]. Migraine most likely influences food choices, and even though some evidence has pointed to a correlation between obesity and migraine [91], this area is in need of further investigation. In this regard, the role of the hypothalamus must be considered. Levels of hypothalamic neurotransmitters, hormones, and adipocytokines have been found to differ in migraine patients. For example, orexin A was higher during the headache phase in migraine, serotonin levels were decreased interictally, insulin resistance was found to be higher in migraine patients, and adipocytokines such as leptin were elevated during migraine [17,92,93].

Considering the successful targeting of CGRP in migraine by novel antibodies [94], one may consider the effects of diet on CGRP, which has been less explored. Only a few experimental studies have reported that some food components alter CGRP expression and secretion levels. Cady and Durham observed significant decreases in the expression of CGRP in rat trigeminal ganglia after eating cocoa-enriched diets for 14 days [95]. In cell-based models, extracts from grape seed, ginger extracts, and petasin present in the botanical butterbur dietary supplement decreased CGRP secretion [96,97].

Collectively, the field of dietary interventions for migraine is awaiting well-designed, systematic, and mechanism-driven research to provide evidence-based dietary recommendations for migraine patients. However, this approach is challenging, expensive, and time-consuming. This also raises awareness that no “migraine diet” *per se* exists, while many diets have shown potential benefit in patients with migraine. In addition, it might be too soon to use some terms such as “epigenetic diet for migraine” [26]. Funding agencies are encouraged to adjust support for nutrition research in migraine to yield reliable outcomes for clinical usage.

Hypothetically, there is always a risk of adverse effects from nutritional deficiencies or excesses, especially if the patient is not being monitored by a healthcare professional. Until evidence-based outcomes become available, attention should be paid to lifestyle and behavioral changes that could be used to prevent or delay the progression of migraine. In addition to recommendations for nutritional arrangements and taking in an adequate amount of daily fluid, adequate sleep hygiene, relaxation and breathing exercises, and the maintenance of a social life are valuable dimensions for lifestyle modification in migraine patients to enhance their overall quality of life.
